# Alteration of the gut fecal microbiome in children living with HIV on antiretroviral therapy in Yaounde, Cameroon

**DOI:** 10.1038/s41598-021-87368-8

**Published:** 2021-04-07

**Authors:** William Baiye Abange, Casey Martin, Aubin Joseph Nanfack, Laeticia Grace Yatchou, Nichole Nusbacher, Clement Assob Nguedia, Hortense Gonsu Kamga, Joseph Fokam, Sean P. Kennedy, Alexis Ndjolo, Catherine Lozupone, Celine Nguefeu Nkenfou

**Affiliations:** 1 Department of Medical Laboratory Sciences, Faculty of Health Sciences, University of Buea, Buea, Cameroon; 2 Medical Microbiology Laboratory, Yaounde University Teaching Hospital, Yaounde, Cameroon; 3 Chantal Biya International Reference Centre for Research on HIV/AIDS Prevention and Management, Yaounde, Cameroon; 4 Department of Medicine, School of Medicine, University of Colorado, Denver, USA; 5 Department of Computational Biology, Institut Pasteur, USR 3756 CNRS, Paris, France; 6 Department of ETN, Faculty of Medicine and Biomedical Sciences, University of Yaounde I, Yaounde, Cameroon; 7 Higher Teachers’ Training College, University of Yaounde I, Yaounde, Cameroon

**Keywords:** Microbial communities, Microbial genetics

## Abstract

Multiple factors, such as immune disruption, prophylactic co-trimoxazole, and antiretroviral therapy, may influence the structure and function of the gut microbiome of children infected with HIV from birth. In order to understand whether HIV infection altered gut microbiome and to relate changes in microbiome structure and function to immune status, virological response and pediatric ART regimens, we characterized the gut microbiome of 87 HIV-infected and 82 non-exposed HIV-negative children from Yaounde, a cosmopolitan city in Cameroon. We found that children living with HIV had significantly lower alpha diversity in their gut microbiome and altered beta diversity that may not be related to CD4+ T cell count or viral load. There was an increased level of *Akkermansia* and *Faecalibacterium* genera and decreased level of *Escherichia* and other *Gamma proteobacteria* in children infected with HIV, among other differences. We noted an effect of ethnicity/geography on observed gut microbiome composition and that children on ritonavir-boosted protease inhibitor (PI/r)-based ART had gut microbiome composition that diverged more from HIV-negative controls compared to those on non-nucleoside reverse-transcriptase inhibitors-based ART. Further studies investigating the role of this altered gut microbiome in increased disease susceptibility are warranted for individuals who acquired HIV via mother-to-child transmission.

## Introduction

The human gastrointestinal tract hosts a complex microbial community (the microbiome) that plays essential homeostatic roles such as energy harvest, pathogen exclusion, and immune regulation^1^. Differences in composition of the microbiome have been shown to affect these functions^2,3^. The microbiome varies considerably between populations, and geography, diet, behavior, and genetic background have all been shown to influence community composition in the gastrointestinal tract^4–6^. HIV infection is characterized by health complications such as metabolic disorders, opportunistic infections, and chronic immune dysfunction, and a growing body of literature has identified the microbiome as an actuator in these processes. However, as most gut microbiome research has been concentrated in industrialized populations, the current understanding of structural and functional variation of microbiomes in developing nations is quite limited, especially in the context of endemic and epidemic diseases and developing health infrastructure. The generalizability of findings in industrial populations to developing ones is unknown, and expanding research in underrepresented populations, especially sub-Saharan Africa (SSA), remains a scientific priority^7^. This will be of paramount importance in SSA, where infectious diseases are prevalent, such as HIV. About 70% of the global AIDS epidemics is concentrated in SSA and anti-retroviral therapy (ART) coverage remains suboptimal in several countries, especially within the pediatric populations^8^. A total of 1.7 million (1.1–2.4 millions) adolescents (aged 10–19) live with HIV worldwide, of which 9/10 live in SSA^8^.

HIV-induced immunodeficiency has been associated with a significant shift in the composition of the gut microbiome, which in turn contributes to HIV disease progression^9–11^. These compositions are sometimes characterized by decreases in community complexity, and increases in proinflammatory bacteria indicative of dysbiosis, particularly in the case of progressive disease and low CD4+ T cell counts^12^. Strategies to inhibit the development of gut dysbiosis during chronic HIV infection and disease progression are especially important in vertical transmission, since, barring a cure, these individuals will be chronically infected with HIV and will require specialized long-term medical care, including antibiotic prophylaxis and ART. Despite the benefits of ART in controlling HIV infection, gut dysbiosis persists even among ART-experienced patients and may contribute to immune impairment, disease progression and inflammatory reaction^13^. However, observations in a pediatric Zimbabwean cohort found that children who had undergone ART for more than 10 years had higher alpha diversity compared to their HIV infected peers. This led to Flygel et al. to propose that prolonged ART could restore microbiome richness^14^.

Though advances in public health initiatives in Cameroon have led to increased survival into adolescence, HIV-positive children are more susceptible to adverse health outcomes. Over 62,000 Cameroonian children live with HIV, representing an important public health demographic in Cameroon that requires specific medical attention (CNLS-Comité National de Lutte contre le Sida), 2018, unpublished data). In 2011, 4 years subsequent to the launch of the Early Infant Diagnosis program, more than 100,000 infants had been tested for HIV infection by PCR, with an 11.5% HIV positive rate. This rate dropped to 9.34% in 2013 and was further reduced to 5.8% in 2018 (15, 16, CNLS, 2018, unpublished data). Early infant diagnosis (EID) in 2018 ensured 96.7% of testing coverage for infants/children enrolled for the prevention of mother-to-child transmission (PMTCT) program (CNLS, 2018, unpublished data). At that time, national pediatric ART coverage was 25.36% (15,927/62,405), of which 8.3% were on ritonavir-boosted protease inhibitor (PI/r)-based ART and 91.7% were on non-nucleoside reverse-transcriptase inhibitors (NNRTI)-based ART (CNLS, 2018, unpublished data). Virological suppression with ART, defined as plasma viral load < 1000 copies/mL, was reported in 76.2% of children aged less than 10 years and 54.5% of adolescents aged 10–19 years old^17^. This poor ART response in pediatric patients could be partly attributed to the high burden of pretreatment drug resistance (48.8%) observed at early age. However, no microbial analysis was carried out, and it is unknown if these children displayed any signs of dysbiosis.

Using culture-based techniques, we previously observed that HIV-infected children on ART and prophylactic co-trimoxazole harbored more abnormal/proinflammatory gut pathobionts (*E. coli*, *Streptococcus spp., Shigella spp., Staphylococcus aureus, Klebsiella spp., Acinetobacter spp., Pseudomonas spp., Clostridium spp, *and *Proteus spp.*) compared to uninfected peers^18^. We therefore postulated that children living with HIV in Cameroon would have a low-diversity dysbiotic gut microbiome when broadly surveyed with 16S ribosomal RNA (rRNA) sequencing, and that community richness would be positively impacted by the duration and efficacy of ART regimen. In this study, we describe the gut microbiome composition of both HIV non-infected (unexposed) and HIV-infected children in Cameroon, and relate changes in microbiome structure and function to immune status, virological response and pediatric ART regimens.

## Results

### Demographic and clinical characteristics of study participants

**Figure 1:**
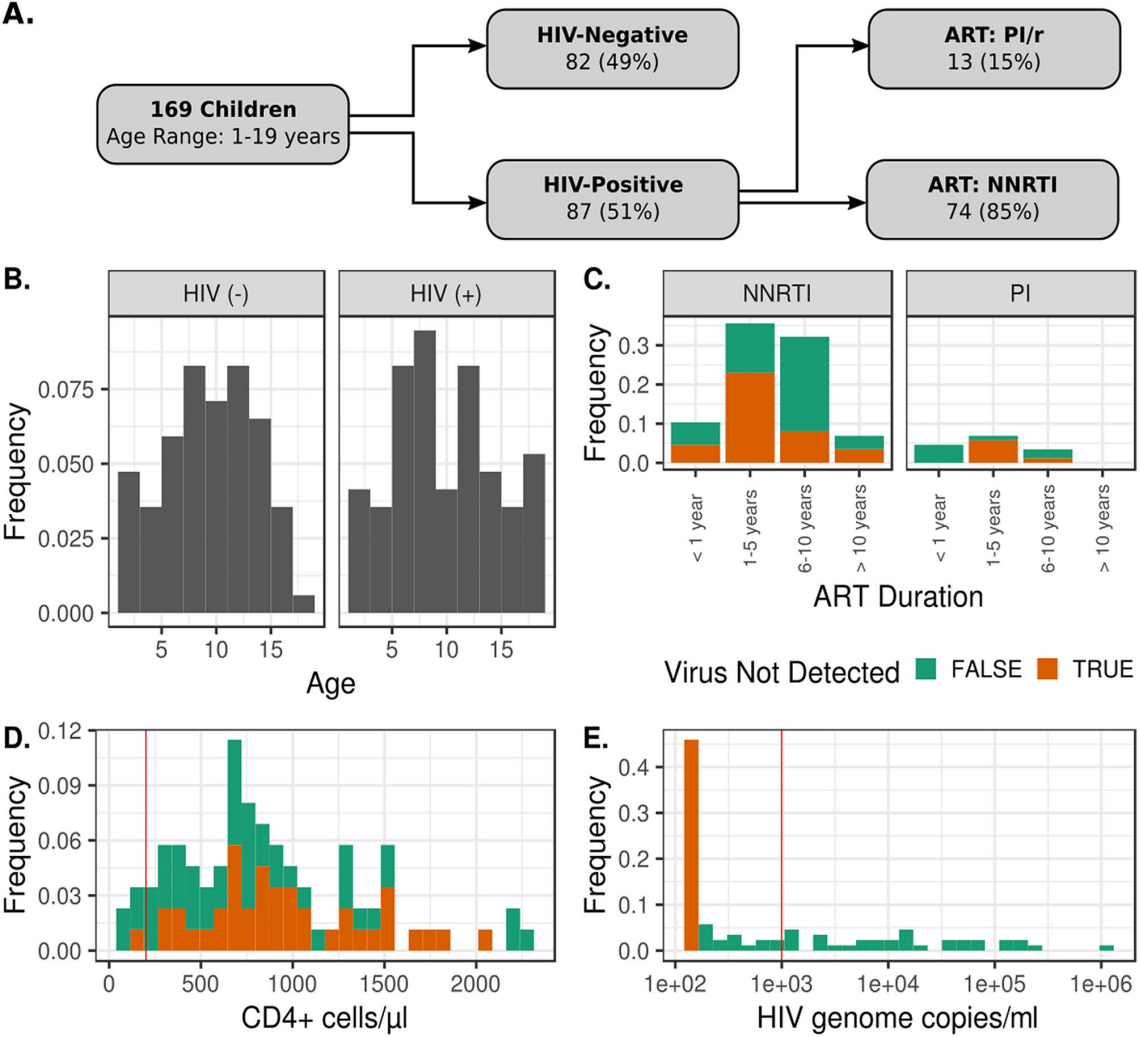
Cohort demographics. (**A**) Gives the breakdown by HIV status and ART regimen. (**B**–**E**) The distributions of participant age, duration of ART treatment, CD4 concentrations, and HIV titers. Vertical red lines depict clinical thresholds of severe immunodeficiency (> 200 cells/ mm^3^) and viremic control (> 1000 genome copies/mL). Limit of detection for HIV genomes is 150 genomes per mL.

The demographic and clinical characteristics of our study population are summarized in Table 1. The flow chart presented in Fig. 1 summarizes the enrolled participants and their clinical parameters. We enrolled 169 participants from January to October 2018. The cohort consisted of 87 HIV-infected participants (51.5%) and 82 non-exposed HIV-negative control participants (48.5%). Individuals were aged between 1 and 19 years with a mean age of 10.13 years. There were no significant differences observed in sex ratio or age category in the HIV-infected versus HIV non-infected group (Table 1). All of the 87 enrolled HIV-infected children were prescribed an ART regimen and co-trimoxazole (250 mg) prophylaxis was initiated once diagnosis was confirmed as recommended by Cameroon’s 2015 national guidelines on the prevention and management of HIV. Overall median [IQR] duration on ART was 5 years with a range of 6 months to 12 years. A majority, 85% of the children, were on an NNRTI-based regimen and the rest were on PI/r (Table 1). Immune status, namely CD4+ T cell counts, was also measured: 5 (5.7%) infected children had CD4+  < 200 cells/ mm^3^, 18 (20.7%) had CD4+ between 200 and 499 cells/ mm^3^, and 64 (73.6%) had CD4+  > 500 cells/mm^3^. Thirteen children (15%) were viremic (plasma viral load > 1000 copies/mL).

**Table 1:** Cohort demographics.

	Overall population sequenced N (%)	HIV-infected children N (%)	HIV non-infected children N (%)	*P* value
# of participants	169	87 (51.5)	82 (48.5)	
**Sex**				
Female	84 (50.6)	44 (4.9)	40 (48.8)	0.878
Male	85 (49.4)	43 (52.1)	42 (51.2)	
**Age**				
1–3 years	15 (8.9)	07 (8.1)	08 (9.8)	0.700
4–6 years	24 (14.2)	15 (17.2)	09 (11)	
7–9 years	42 (24.9)	21 (24.1)	21 (25.6)	
≥ 10 years	88 (52.0)	44 (50.6)	44 (53.6)	
**CD4+ cells/mm**^**3**^				
< 200	–	5 (5.7)	NA	
200–499	–	18 (20.7)	NA	
≥ 500	–	64 (73.6)	NA	
**Viral load copies/mL**				
Not detected	–	40 (45.9)	NA	
< 150–1000	–	15 (17.2)	NA	
1001–10,000	–	13 (14.9)	NA	
> 10,000	–	19 (21.8)	NA	
**ART regimens**				
Pis	–	13 (14.9)	NA	
NNRTIs	–	74 (85.1)	NA	
**ART duration**				
< 1 year	–	13 (14.9)	NA	
1–5 years	–	37 (42.5)	NA	
6–10 years	–	31 (35.6)	NA	
> 10 years	–	6 (7.0)	NA

### Description of microbiota in Cameroonian children

**Figure 2:**
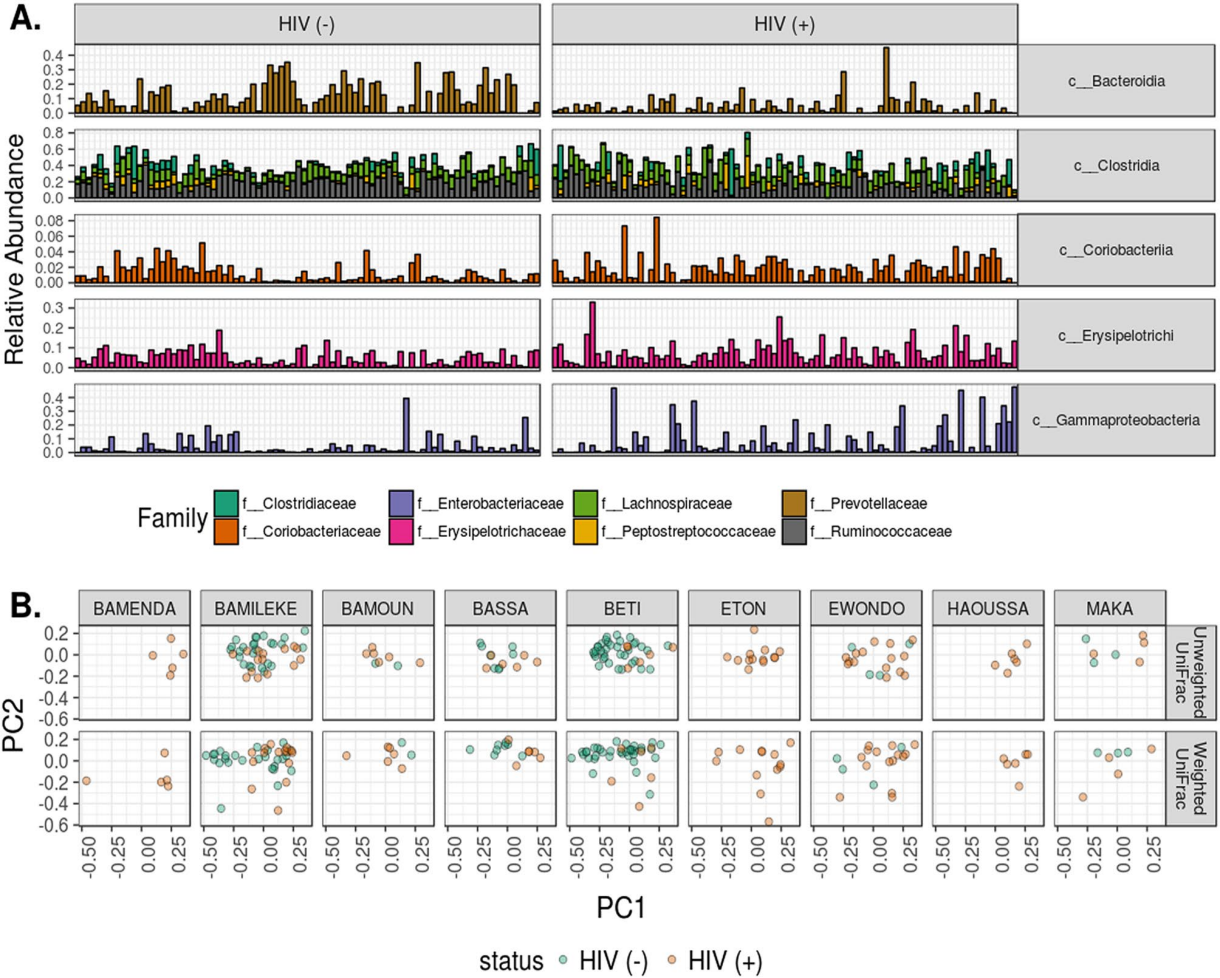
Taxonomic and compositional description of microbiomes in Cameroonian children. In (**A**), the taxonomic composition at the class and family levels is given for prevalent lineages (ASVs present in at least 20% of samples). Bacterial families with a median relative abundance less than 1% were removed for ease of visualization. (**B**) The PCoA decomposition of unweighted and weighted UniFrac distances for ethnic groups with greater than 5 samples. When controlling for HIV infection status, ADONIS analysis showed differential clustering by ethnicity (*p* < 0.05).

The overall analysis of 16S rDNA data identified a total of 3,968 amplicon sequence variants (ASVs). Study participants had microbiomes relatively rich in the genus Prevotella and poor in Bacteroides, similar to other studies conducted in SSA^10^ (Fig. 2). We used ADONIS testing to detect compositional differences in microbiome structure. This test is a permutational multivariate analysis that assesses the group-wise, phylogenetic clustering of microbiomes. When controlling for HIV status, ADONIS testing of weighted and unweighted UniFrac distances revealed compositional differences by ethnicity (*p* < 0.05), excluding Bamenda, Eton, and Haoussa ethnic groups as there were no HIV negative representatives belonging to these ethnic groups, indicating that there are population-level differences in microbiome structure.

### HIV infection is associated with decreased microbial diversity and changes in community structure

**Figure 3:**
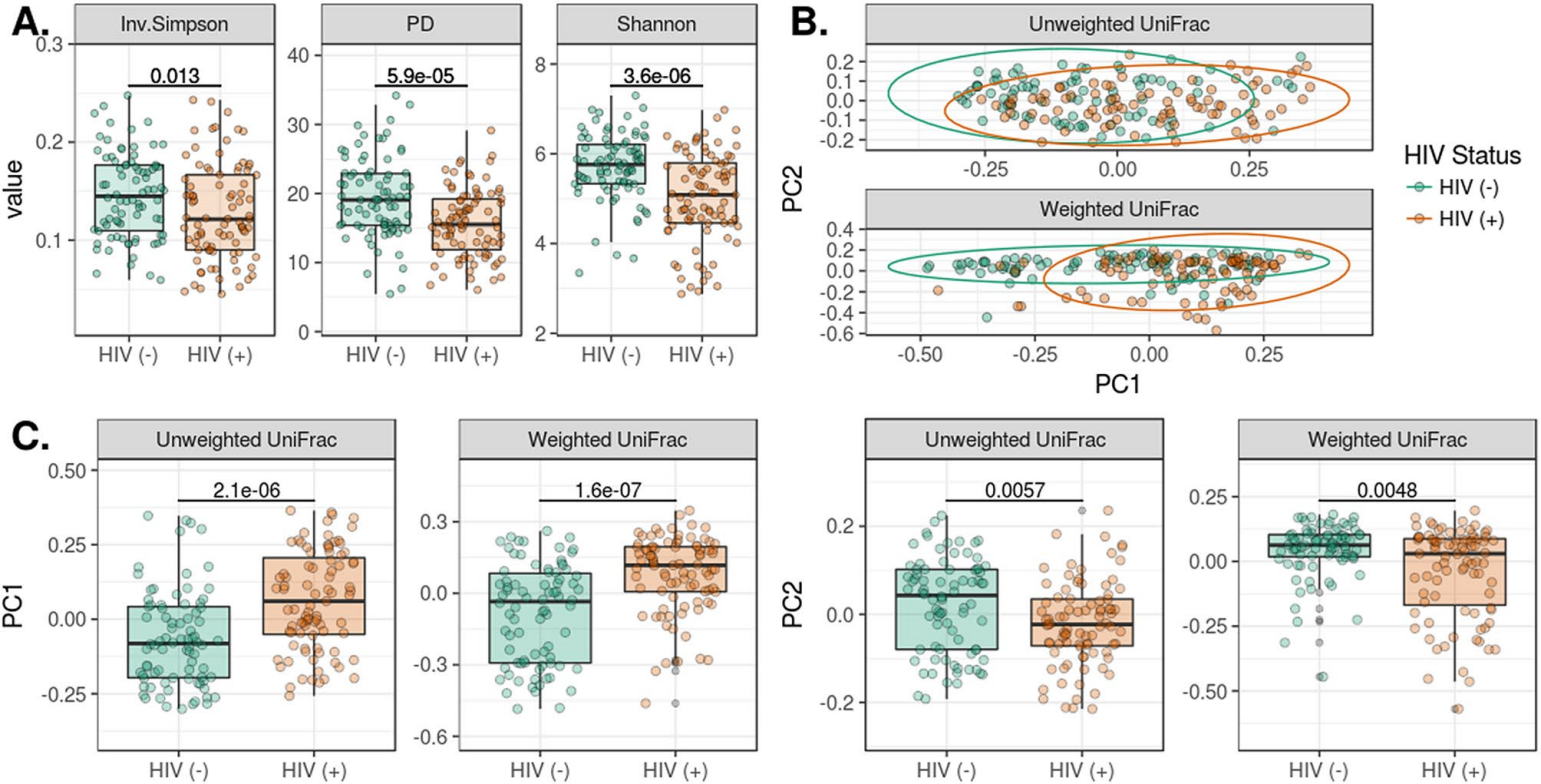
Changes in alpha and beta diversity by HIV infection status. In (**A**), we show that HIV infection and treatment were associated with decreases in community diversity using the Inverse Simpson, Shannon, and phylogenetic diversity (Faith’s PD) measures. (**B**) Plots the PCoA for the first two principal coordinates of weighted and unweighted UniFrac distances. ADONIS testing revealed differences in clustering by HIV status for both weighted and unweighted measures (p < 1e−5). Differences in bacterial composition between HIV-infected and HIV non-infected are captured in the first two principal coordinates as shown in (**C**).

**Figure 4:**
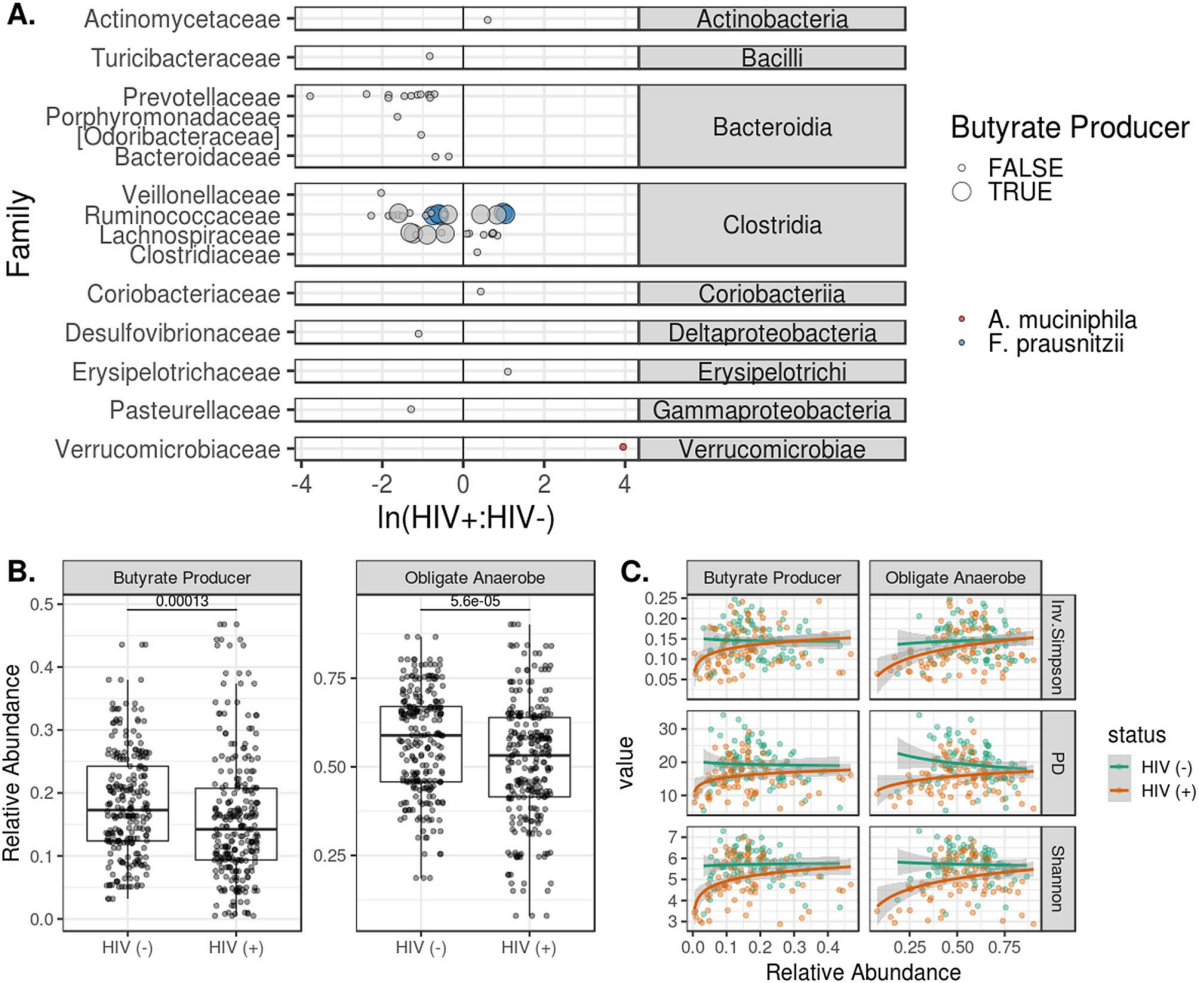
Differential abundance of ASVs and microbiome function by HIV status. Differentially represented ASVs in the HIV infected and HIV non infected cohorts were identified via paired, Wilcoxon tests. Mean relative abundances were calculated for these ASVs for the HIV-negative and positive cohorts. In (**A**), the log-ratio of those mean abundances are plotted on the x axis, with ASVs more abundant in the HIV infected cohort having a ratio greater than 0. ASVs are grouped at the class level, and the family-level taxonomic assignment is given on the y axis. ASVs that were predicted to produce butyrate are shown as large circles, while small circles donate ASVs that are not capable of butyrate production. ASVs mapped to *A. muciniphila* and *F. prausnitzii* are colored red and blue, respectively. ASVs with genus-level taxonomic assignments were filtered against the “List of Prokaryotes according to their Aerotolerant or Obligate Anaerobic Metabolism” and were assigned a binary value for predicted butyrate production and obligate anaerobic growth. Relative abundances for ASVs predicted to have these traits are shown in (**B**). There were differing relative abundances of butyrate producers and obligate anaerobes by HIV status (p < 0.001). (**C**) Depicts the log-linear relationships between the relative abundance of these traits and the corresponding alpha diversity (p < 0.001).

In this study, we used three metrics to gauge alpha diversity: Inverse Simpson (evenness), Faith’s (phylogenetic diversity) and Shannon Index (richness and evenness) (Fig. 3A). HIV infection and treatment was associated with decreases in community diversity with all three measures (Fig. 3A). ADONIS testing of unweighted and weighted UniFrac distances indicated compositional differences by infection status (*p* < 0.001). We used Principal Coordinate Analysis (PcoA) decomposition to visualize clustering by community structure (Fig. 3B), and then verified that the first two principal coordinates of the spatial decomposition captured variance between the HIV infected and HIV non infected groups (Fig. 3C). To resolve which bacterial strains were driving these changes, we identified ASVs that were differentially abundant by HIV status. After filtering rare and low-abundance taxa, we used Wilcoxon tests to identify 87 ASVs that differed in children living with HIV when compared to healthy controls (Fig. 4A). Two-thirds of the ASVs, were under-represented in children living with HIV (Fig. 4A). Using the “List of Prokaryotes according to their Aerotolerant or Obligate Anaerobic Metabolism”, we estimated the relative fraction of bacteria that were capable of butyrate production and were obligate anaerobes. We found that the HIV infected cohort had lower relative fractions of butyrate producers and obligate anaerobes (*p* < 0.05) (Fig. 4B). Both of these estimates of function were predictive of alpha diversity measures (*p* < 5e−8) with differing responses by disease status (*p* < 0.001) (Fig. 4C). Children living with HIV showed a strong drop in alpha diversity at low fractions of butyrate producers and anaerobes when compared to their HIV non infected counterparts.

### CD4+ T cell concentrations and viral titer are neither predictive of microbiota diversity nor each other

Individuals were binned by CD4+ T cell counts into HIV-negative, ≤ 200, and > 200 cells/ mm^3^ groups. No changes in alpha diversity were observed in the microbial communities for participants with CD4+ T cell counts ≤ 200 cells/ mm^3^ compared to those with > 200 cells/mm^3^ (Figure S1, panels A and C). Linear modeling of CD4+ T cell counts and alpha diversity also did not reveal any relationships. A similar schema was used for our analysis of the relationship between viral titers and alpha diversity. Viral load values were binned into three groups: HIV non-infected, < 1000, and ≥ 1000 copies/mL and comparison of alpha diversity metrics did not reveal viral burden-based differences. Linear modeling of viral titers and alpha diversity metrics also had null findings (Figure S1, panels B and D). ADONIS testing of UniFrac distances indicated no differences in community composition by immune status or viral burden categories. CD4+ T cell counts and viral load were also not correlated with each other.

### Alpha and beta diversity varies with ART regimen but not duration

**Figure 5:**
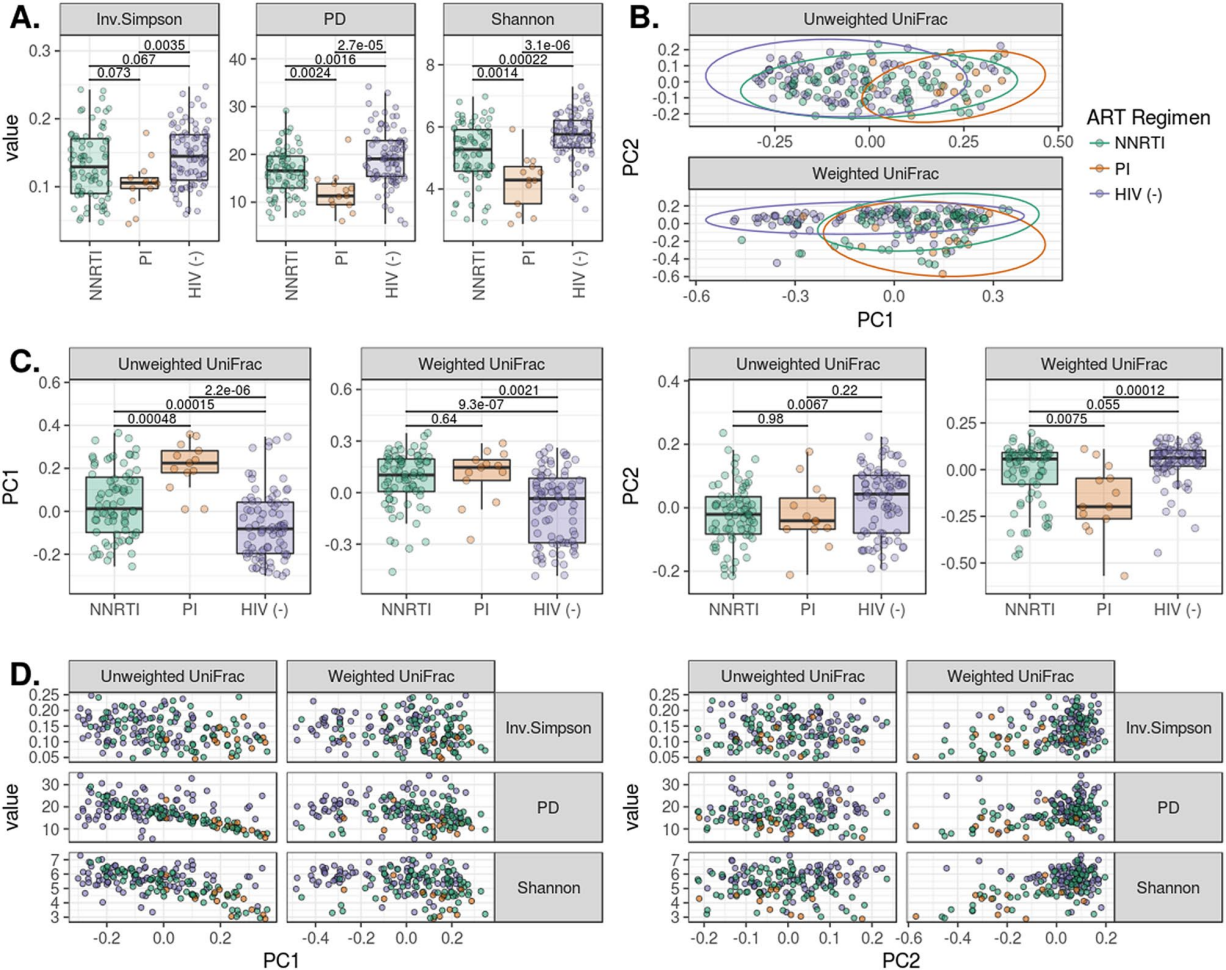
ART regimen-specific effects on diversity and composition. (**A**) Shows that PI treatment is associated with reduced bacterial evenness and richness when compared to HIV non-infected individuals and those on NNRTI regimens. ADONIS testing of weighted and unweighted UniFrac distances indicated differential clustering when comparing NNRTI to PI-based regimens (*p* < 0.05). Differences in ordination of the UniFrac distances by ART regimen are detailed in (**C**). (**D**) Depicts the relationships between UniFrac ordinations and alpha diversity.

**Figure 6:**
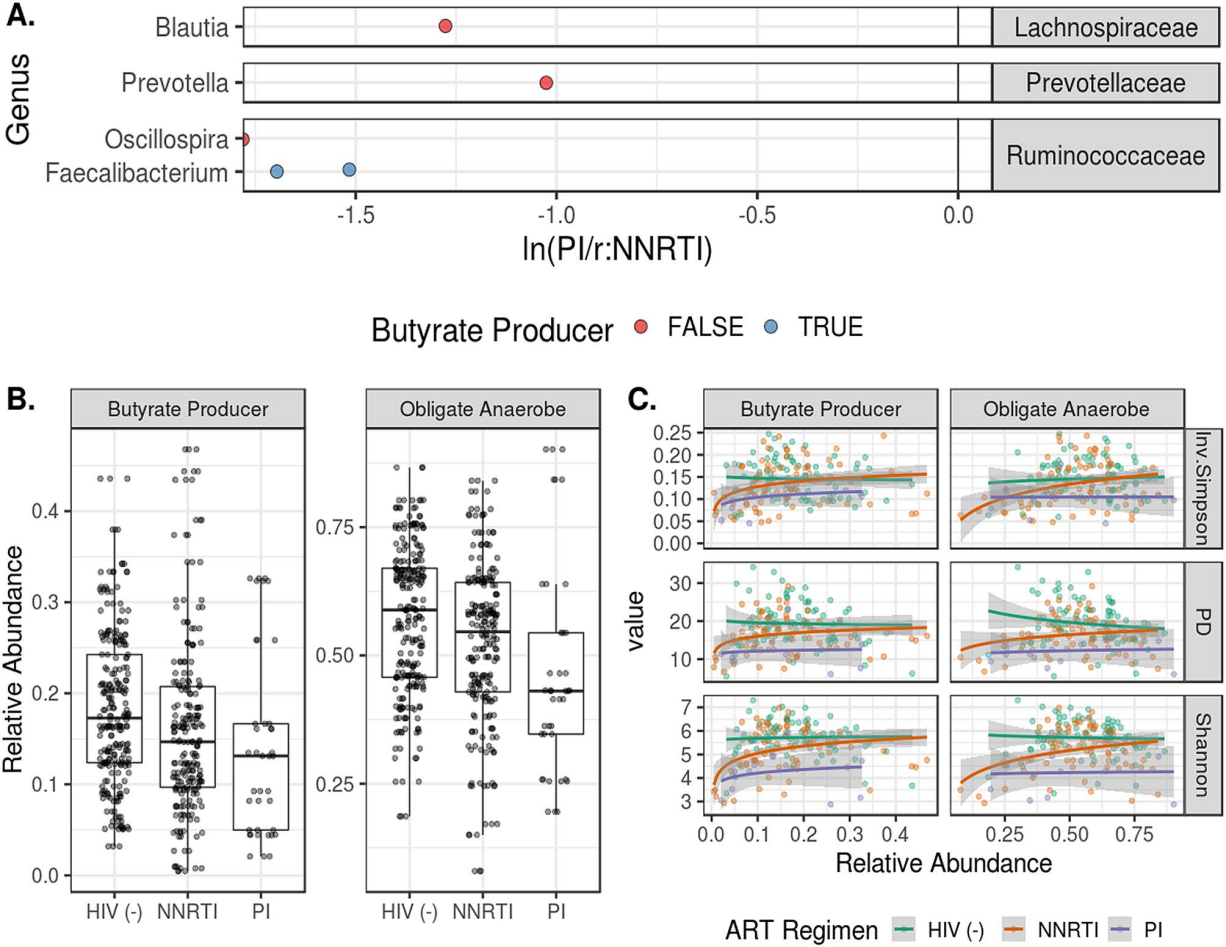
Differential abundance of ASVs and microbiome function by ART regimen. Differentially represented ASVs in the HIV-positive NNRTI and PI/r cohorts were identified via paired, Wilcoxon tests. Mean relative abundances were calculated for these ASVs for the NNRTI and PI/r cohorts. In (**A**), the log-ratio of those mean abundances are plotted on the x axis, with ASVs less abundant in the PI/r cohort having a ratio less than 0. ASVs are grouped at the family level, and the genus-level taxonomic assignment is given on the y axis. ASVs with genus-level taxonomic assignments were filtered against the “List of Prokaryotes according to their Aerotolerant or Obligate Anaerobic Metabolism” and were assigned a binary value for predicted butyrate production and obligate anaerobic growth. Relative abundances for ASVs predicted to have these traits are shown in (**B**). There was a trending difference relative abundances of butyrate producers and obligate anaerobes by HIV status (p = 0.06). (**C**) Depicts the log-linear relationships between the relative abundance of these traits and the corresponding alpha diversity (p < 5e−8).

Children on ritonavir-boosted protease inhibitor (PI/r)-based ART had lower alpha diversities and altered composition when compared to children on NNRTI-based ART as well as their HIV non-infected counterparts (Fig. 5). The compositional changes are captured in the first principal coordinate in the Unweighted Unifrac PCoA and in the second principal coordinate for weighted UniFrac. Using linear models, we were unable to detect a relationship between alpha-diversity and ART duration. Increasing model complexity by controlling for age, CD4 count, and viral burden did not improve predictive power. (Figure S2). We observed differential ASV distribution according to ART regimen with ASVs classified in the Blautia, Prevotella, Oscillospira and Faecalibacterium genera more abundant in the NNRTI cohort compared to PI/r cohort (Fig. 6A). These shifts in composition showed a trend in differing representation of butyrate producers and obligate anaerobes by ART regimen (p = 0.06) with PI/r treated individuals displaying a larger loss in organisms predicted to have these functions (Fig. 6B). Loss of butyrate producers and obligate anaerobes was associated with a decrease in alpha diversity measures (p < 5e−10) with varying responses by ART regimen (p < 0.01) (Fig. 6C). At commensurate levels of butyrate producers and obligate anaerobes, the PI/r cohort had lower alpha diversity than the HIV non-infected and NNRTI cohorts, while the individuals taking NNRTI had an intermediate response.

## Discussion

In this study we characterize the gut microbiome in a pediatric population of ethnically diverse Cameroonian children with and without HIV. Children living in Cameroon and in SSA broadly have been underrepresented in studies of the gut microbiome, with studies conducted in SSA often focusing on populations with specific lifestyles such as agrarian, pastoralist, or hunter-gathers^4,19–26^. However, one study conducted in individuals in three populations along an urbanization gradient in Cameroon indicated that urban individuals have retained most of their gut eukaryotic and bacterial diversity despite significant changes in diet compared to the rural areas^27^. Our results are consistent with this work, with the ethnically diverse Cameroonian children surveyed here harboring a microbiota characteristic of agrarian-associated enterotypes with communities characterized by high Prevotella and low Bacteroides and various lineages of *Clostridia*, regardless of HIV-infection status. When controlling for HIV treatment status, we identified differences in microbial composition among Cameroonian ethnic groups. It is unknown how these compositional differences are driven by genetic background, diet, and environmental exposures. In Cameroon, customary food staples vary across and within ethnic groups as the preferences for one staple over another are influenced by cultural history/identity and geography^28^. Though some research has investigated the link between subsistence lifestyle and microbiome composition in rural Cameroon^19^, much remains to be discovered about how the interplay between cultural identity, geography, and diet influences microbiome structure and function across Cameroon’s ~ 250 ethnic groups. Unfortunately, during the present study, diet data specific to each community was not collected, and thus constitute a limitation of this study.

Children living with HIV in Cameroon represent a priority study population because they experience higher rates of other infections and adverse health events^29^. We identified decreased alpha diversity and shifts in community structure in HIV-infected children in Cameroon. This decrease in alpha diversity was not related to low CD4+ T cell counts as has been previously described in a cohort in Uganda^30^. The gut microbiome of HIV-infected children was also characterized largely by a depletion of *Prevotella* and *Clostridia* and an increase in other microbes such as *Akkermansia muciniphila* and *Faecalibacterium prausnitzii*. Overall, estimates of the prevalence of obligate anaerobes and butyrate producers indicate that HIV infection and treatment results in a loss of some key microbiome parameters. Because the children living with HIV were receiving treatment with both ART and prophylactic co-trimoxazole, we cannot differentiate how these drugs individually impact diversity. Although it is tempting to speculate that the reduced alpha diversity observed is driven by long term antibiotic use, one randomized controlled study conducted in HIV-exposed uninfected children in SSA indicated that co-trimoxazole actually increased alpha diversity^31^.

In this cohort, compositional shifts in HIV infection and treatment were characterized by a marked decrease in *Prevotella*. Early investigations in HIV-associated microbiomes reported increases in the *Prevotella* genus in HIV infected Western populations, but these findings were subsequently shown to be found in high-risk Men who have Sex with Men (MSM) independently of HIV infection status^9,10^. The identification of *Prevotella* strains and their functional differences among different HIV-infected demographics is still an area of active research^19,32–36^. Since our HIV-infected cohort is on ART, the decrease in *Prevotella* that we observed here is perhaps consistent with a prior study has found a decrease in *Prevotella* in HIV-infected individuals with ART^37^.

*Akkermansia* and *Faecalibacterium* are two notable genera that were increased in children living with HIV, an inverse relationship to observations made in other studies^38^. We also observed decreased levels of *Escherichia* and other Gammaproteobacteria in children living with HIV which is counter to observations made in studies in western populations^39,40^. In contrast to another study where both *Akkermansia muciniphila* and *Faecalibacterium prausnitzii* were depleted with HIV infection^39^, we saw an increase in both taxa in our HIV-infected cohort. *Faecalibacterium prausnitzii* is a commensal butyrate producer that is commonly lost in oxygen-rich, inflammatory, low-diversity dysbiotic states and has been the subject of many probiotic investigations^41^. *F. prausnitzii* is a fastidious, obligate anaerobe, and its retention in the gut despite HIV infection and treatment suggests maintenance of a hypoxic environment in the intestine, a key chemical criterion for host-microbe homeostasis. *Akkermansia muciniphila*, another popular probiotic candidate, shares trophic chains with *F. prausnitzii* and stimulates butyrate production when co-cultured in vitro^42^. *Akkermansia* resides in the mucus layer of the large intestine, where it is directly involved in modulating intestinal integrity^43,44^ and is also capable of engaging in mutualistic interactions with butyrate-producing bacteria in its local physiological niche^42^. This metabolic enmeshment with the host and other host-adapted commensals has led to the hypothesis that *Akkermansia* may represent a keystone species in the human gut^45,46^. In addition to the observation of increased prevalence of these beneficial bacteria with HIV, we also found that certain gammaproteobacteria, *Haemophilus influenzae* and an unassigned Enterobacteriaceae member, were decreased in children living with HIV in Cameroon, which is in contrast to previous reports in HIV-infected populations^47^. Taken together, these results suggest that certain aspects of the HIV-associated microbiome in these children would indicate a healthier microbiome. One potential factor that may explain this surprising observation is the reported beneficial effects of co-trimoxazole on inflammation. Specifically, prior randomized controlled studies conducted in HIV-exposed HIV negative and/or HIV-infected children in SSA have found that terminating co-trimoxazole treatment increased systemic inflammatory markers^48^ and was associated with increased prevalence of pro-inflammatory Streptococci^48,49^. Furthermore, Bourke et al. went on to demonstrate in an in vitro study that co-trimoxazole reduces intestinal inflammation by blunting immune and epithelial cell activation directly^48^. Another study in Burkina Faso administered co-trimoxazole to healthy children and observed weak changes in diversity when compared to healthy controls, though they did not specify which bacteria were influenced^50^. These effects may help for beneficial mucosa-associated bacteria, such as *Akkermansia*, to establish and further supports use of prophylactic co-trimoxazole in this population. As the current body of literature suggests that co-trimoxazole exerts minimal influence on the microbiome in health and has restorative characteristics in disease, we hypothesize that the observed loss of diversity is either attributed to ART and/or HIV infection, or is driven by use of co-trimoxazole during very early life, a critical window for microbiome development.

Advanced HIV infection largely destroys the intestinal CD4+ T cell population and can induce severe mucosal inflammation^51^. This leads to loss of epithelial membrane integrity and translocation of microbes and their products into the lamina propria and eventually into systemic circulation. This loss of compartmentalization correlates negatively with microbial diversity^52^. However, we found no association between microbial diversity and circulating CD4+ T cell counts or viral load. This is in contrast to studies by Monaco et al.^52^ and Ribeiro et al.^53^, who reported decreased diversity in patients with CD4 < 200 cells/ mm^3^, though it is possible that a larger number of immune-compromised individuals is necessary to detect these signals, as only 5 (5.7%) of our participants were severely immunocompromised (CD4 < 200cells/ mm^3^). If community composition, diversity and immune status are indeed linked, it is conceivable that the low diversity dysbiosis is contingent upon immune dysfunction over longer timescales or at greater disease severity than those observed in this cross-sectional cohort. It is also possible that this cohort has attributes (e.g. diet, environmental exposure, baseline microbiome composition) that confer a resilience to dysbiotic shifts during perturbations like immune dysfunction. Also, in our study we did not evaluate other markers of immune dysfunction that are known to be important in HIV infection, such as immune activation, including increases in HLADR+ CD38+ CD4 and CD8 T cells, and immune exhaustion, characterized by increased expression of the marker PD1 on T cells. Further work would benefit from relating microbiome differences to more in depth immune phenotyping of this population.

We were unable to identify any interactions between ART+ co-trimoxazole treatment duration, age, and microbial community diversity (Figure S2). This suggests that any ART+ co-trimoxazole-mediated changes to the microbiome manifested before sampling. As our cohort included children with less than 1 year of treatment, the drugs’ effects on microbiome dynamics may manifest in the months of initiation and stabilize soon after. The multifactorial nature of HIV management makes it difficult to identify which aspects of disease or treatment are driving the changes in the microbiome. Advances in public health infrastructure in Cameroon have made ART+ antibiotic treatment so widely available that it is difficult to establish an untreated HIV-positive pediatric cohort in an urban center. Although some studies have been performed in which children are randomized to temporarily suspend or continue co-trimoxazole treatment^48,49^; these studies do not address effects of being on co-trimoxazole long term and during early life, a known crucial stage in microbiome development. As public ART coverage efforts in Cameroon continue to grow, expansion to these under-serviced populations may provide an opportunity for researchers to sample children before and after they begin their ART regimens. Such study designs would address the gaps in knowledge of microbiome dynamics during pharmaceutical disturbance in an important development window. Several investigations on the effect of ART on the gut microbiome, mostly in cross-sectional cohorts (^34,38,52,54,55^), found that ART partially reverses HIV-associated gut dysbiosis, but may also lead to alternative, persistent dysbiotic states^56^. Our results are also consistent with those of another study conducted in Mexico that found that PI/r-based regimens had more severe effects than NNRTI-based regimens^54^ on gut microbiome diversity and composition. We identified lower alpha diversity and altered bacterial composition in children undergoing PI/r treatment when compared to others on NNRTI regimens. Protease inhibitors are known to inactivate cytochrome P450 (CYP), an enzyme involved in hepatic xenobiotic metabolism in humans, and off-target effects are not restricted to the human host. Gut commensals such as *Prevotella copri*, *Eubacterium rectale,* and *Rosburia intestinalis* have decreased fitness when cultured in the presence of saquinavir, a weak CYP inhibitor^57^. Similarly, we observed broad decreases in Prevotellaceae and Lachnospiraceae relative abundance in our HIV-infected cohort. We performed BLAST queries of *P. copri, E. rectale, and R. intestinalis* genomes in the NCBI non-reference database and failed to identify any CYP homologs. *E. coli,* which is known to lack CYP homologs, is also inhibited by saquinavir^55^ which suggests that there are CYP-independent mechanisms of action. Bacterial processing of pharmaceuticals has been shown to reduce bioavailability for the host and potentially impede clinical efficacy^58^. Additionally, the secondary products from bacterial xenobiotic metabolism likely impose second-order effects on other members of the microbial community, and the metabolic pathways and ecological contexts that drive these phenomena await discovery.

## Conclusion

Cameroonian children harbored an intestinal microbiota characteristic of agrarian-associated lifestyles and displayed varying baseline compositions with respect to their ethnicity. HIV-infected, ART-treated children were characterized by decreased alpha diversity and shifts in community structure. Some of these changes, such as depletion of Prevotella and Clostridia species and loss of obligate anaerobes and butyrate producers, are canonical signatures of dysbiosis, while others, increased relative abundance of *Akkermansia muciniphila* and *Faecalibacterium prausnitzii*, were unexpected as these taxa are typically thought of as indicative of intestinal health. ART regimen was associated with varying degrees of dysbiosis with PI/r based regimens presenting more severe losses in diversity than NNRTI-based regimens. Though the accompanying changes in function are still unknown, our findings highlight ART-based differences in microbiome structure, and that CD4+ concentrations and plasma viral loads do not appear to have an effect on the microbial diversity in a cohort of immunocompetent children living with HIV.

## Materials and methods

### Ethics statement

This protocol was evaluated and approved by the National Ethics Committee of Cameroon. We conducted this case (HIV-infected) control (HIV non-infected) study according to the approved protocol guidelines. All participants were children, between 1 to 19 years old and had parental written informed consent.

### Enrollment and sample collection

Subjects were enrolled at two collection sites in Yaounde and samples collected were transferred to the International Research Centre for HIV/AIDS Management and Prevention, Chantal Biya’s, Yaounde. Our control group (HIV uninfected as determined by negative ELISA assay) were screened at the entry point at the Pediatric Units at our collection sites. In the HIV-infected cohort, all infected children were on prophylactic co-trimoxazole as from 6 weeks of age, and on ART once diagnosis was confirmed according to the protocol of National guidelines on the prevention and management of HIV in Cameroon. 74 of them were on the NNRTIs: ABC+3TC+EFV (20 participants), ABC+3TC+NVP (20 participants), TDF+3TC+EFV (19 participants), AZT+3TC+EFV (8 participants), and ABC+3TC+NVP (7 participants). All the 13 PIs participants were on the molecules ABC+3TC+LPV/r.

The specifics of the cohort attributes can be found in Table 1. None of the participants were taking antibiotics or antifungal other than the prescribed prophylactic co-trimoxazole at the time of recruitment or within the past 4 weeks prior to sample collection. Socio-demographic and clinical data were collected using a questionnaire.

Fecal samples were collected in a dry, sterile stool container and children who were unable to provide stool the same day were advised on how to collect stool in the provided container and bring them the next day. Samples were transported to the laboratory within 30 min of collection in a cooler box and immediately placed at − 30 °C. Blood samples were collected by venipuncture at the collection sites and transported to the Medical Analyses Laboratory, International Research Centre for HIV/AIDS Management and Prevention, Chantal Biya’s, Yaounde for analysis.

### Plasma viral load and CD4+ T cell counts

HIV plasma viral load was determined by automated real time polymerase chain reaction (PCR) using the Abbott Real Time HIV-1 platform with a detection limit of 40 copies of HIV RNA copies/mL for 600µL plasma input or 150 copies/mL for a 200 µL plasma input. CD4+ T cell counts were obtained by flow cytometry using the BD FACSCalibur at the Medical Analysis Laboratory, International Research Centre for HIV/AIDS Management and Prevention, Chantal Biya’s, Yaounde.

### DNA extraction from fecal samples

Fecal DNA was extracted at the System Biology Laboratory at the International Research Centre for HIV/AIDS Management and Prevention, Chantal Biya’s, Yaounde using PowerSoil DNA Isolation Kit according to the manufacturer instructions. Extracted DNA was transported frozen to the Lozupone lab at the University of Colorado, Anschutz Medical Campus Aurora Colorado for subsequent processing. Bacterial DNA concentration and purity was evaluated on a Nanodrop N1000 (Thermo Fisher Scientific, Carlsbad, CA, USA) by measuring the A260/A280 ratio. DNAs with an A260/A280 ratio of 1.8–2.0 were used for PCR amplification.

### 16S rRNA targeted sequencing, processing and analysis

The V4 region (515f.-806r; FWD: GTGCCAGCMGCCGCGGTAA; REV: GGACTACHVGGGTWTCTAAT) of the 16S rDNA gene was amplified using 5 PRIME Hot Master Taq DNA polymerase (Quantabio). Primer construction and amplification followed the Earth Microbiome Project (www.earthmicrobiome.org) protocol. Amplified barcoded DNA fragments were quantified using a PicoGreen assay (Invitrogen) and equal amounts (ng) of DNA from each sample were pooled. The aggregate pool was sequenced using a V2 2 × 250 kit on the Illumina MiSeq platform (San Diego, CA) at the University of Colorado Cancer Center, Genomics and Microarray Core Facility. 16S rRNA amplicons were demultiplexed using QIIME 1.9.0 and then denoised into ASVs using DADA2. QIIME2 was then used to rarefy to 11,233 sequences per sample, and calculate alpha diversity (Shannon, Faith’s PD, and Inverse Simpson’s) and beta diversity measures (weighted and unweighted UniFrac). ASVs with genus-level taxonomic assignments were filtered against the “List of Prokaryotes according to their Aerotolerant or Obligate Anaerobic Metabolism” and were assigned a binary value for predicted butyrate production and obligate anaerobic growth. Relative abundances for butyrate production and obligate anaerobic growth were calculated by multiplying the binary value by the relative abundance for the microbe within that sample.

### Statistical analysis

Data were processed using the “tidyverse” ecosystem^59^. Pairwise comparisons of alpha diversities, principal coordinates of UniFrac distances, and trait abundances were performed in R, version 3.4.4^60^ using the Wilcoxon test with Benjamini–Hochberg multiple test correction. To test for differentially represented microbes by HIV status, we first filtered for ASVs which were present in at least 15% of the samples in our population and then compared relative abundances using the Wilcoxon test with Benjamini–Hochberg test corrections. A similar framework was used for differential ASV abundance between the NNRTI and PI/r cohorts, except with a prevalence threshold of 30% instead of 15%. Microbiome clustering by UniFrac dissimilarity was measured using the ADONIS test from the “vegan” package^61^ with 1000 permutations. When analyzing differential beta-diversity by ethnicity (ethnicity ~ *Xβ*) where *Xβ* is the UniFrac distance matrix, only HIV-negative individuals were included, and ethnic groups with less than 5 HIV-negative individuals were omitted for statistical power. UniFrac clustering by HIV status and ART regimen was performed on the whole cohort using the same test parameters. We attempted to model alpha diversity (α) as a function of ART duration which was rounded to the nearest year (*α* ~ *art_duration*). We also created expanded models that incorporated immunological and viremic parameters (*α* ~ *art_duration* + *viral_load* + *cd4_count*). To detect relationships between between butyrate production, obligate anaerobiosis, and α by disease status, we created models *α* ~ *ln(butyrate)* + *HIV_status* and *α* ~ *ln(anaerobe)* + *HIV_status* where ln(butyrate) and ln(anaerobe) are the natural logs of the relative fractions of ASVs with the butyrate or anaerobiosis traits. We used R’s linear model, “lm”, to create and assess the predictive power of the model and contribution of the individual parameters. Data was visualized with “ggplot2”, “cowplot”, “gridExtra”, and “ggsignif”^62–65^.

## Supplementary Information

Supplementary Information

## Data Availability

16S rRNA sequence data is under submission at the European Nucleotide Archive (ENA).
