# Supplementary material for: Alteration of the gut fecal microbiome in children living with HIV on antiretroviral therapy in Yaounde, Cameroon
Source: Sci Rep. 2021 Apr 7;11:7666. doi: 10.1038/s41598-021-87368-8 (PMC8027858; doi:10.1038/s41598-021-87368-8)
Supplement: Supplementary file 1 — Supplementary Information [file 41598_2021_87368_MOESM1_ESM.docx]

**ALTERATION OF THE GUT FECAL MICROBIOME IN CHILDREN LIVING WITH HIV ON ANTIRETROVIRAL THERAPY IN YAOUNDE, CAMEROON**

William Baiye Abange,^1,2,3ø^ Casey Martin,^4ø^ Aubin Joseph Nanfack,^3^ Laeticia Grace Yatchou,^3^ Nichole Nusbacher,^4^ Clement Assob Nguedia,^1^ Hortense Gonsu Kamga,^2^ Joseph Fokam,^3^ Sean P Kennedy,^5^ Alexis Ndjolo,^3,6^ Catherine Lozupone,^4#^ Celine Nguefeu Nkenfou^3,7#^*

^1^ Department of Medical Laboratory Sciences, Faculty of Health Sciences, University of Buea, Cameroon

^2^ Medical Microbiology Laboratory, Yaounde University Teaching Hospital, Cameroon

^3^ Chantal Biya International Reference centre for research on HIV/AIDS prevention and management, Yaounde, Cameroon

^4^ Department of Medicine, School of Medicine, University of Colorado, Denver, USA.

^5^ Department of Computational Biology, Institut Pasteur, USR 3756 CNRS, Paris, France

^6^Department of ETN, Faculty of Medicine and Biomedical Sciences, University of Yaounde I, Cameroon

^7^Higher Teachers’ Training College, University of Yaounde I, Yaounde, Cameroon

^ø^The two authors contributed equally

^#^The two authors contributed equally

Running title: Fecal microbiome in HIV treated Cameroonian children

*Corresponding author: nkenfou@yahoo.com

**Supplementary Figures:**

**Figure S1: Immune status and viral burden are not predictive of bacterial diversity.** Panels A and B show alpha diversities for individuals at different states of immune integrity and viremic control, respectively, with no differences found within the HIV-infected group. Panels C and D show the same readouts as continuous variables; no statistical interactions between these patient parameters and alpha-diversity were detected.


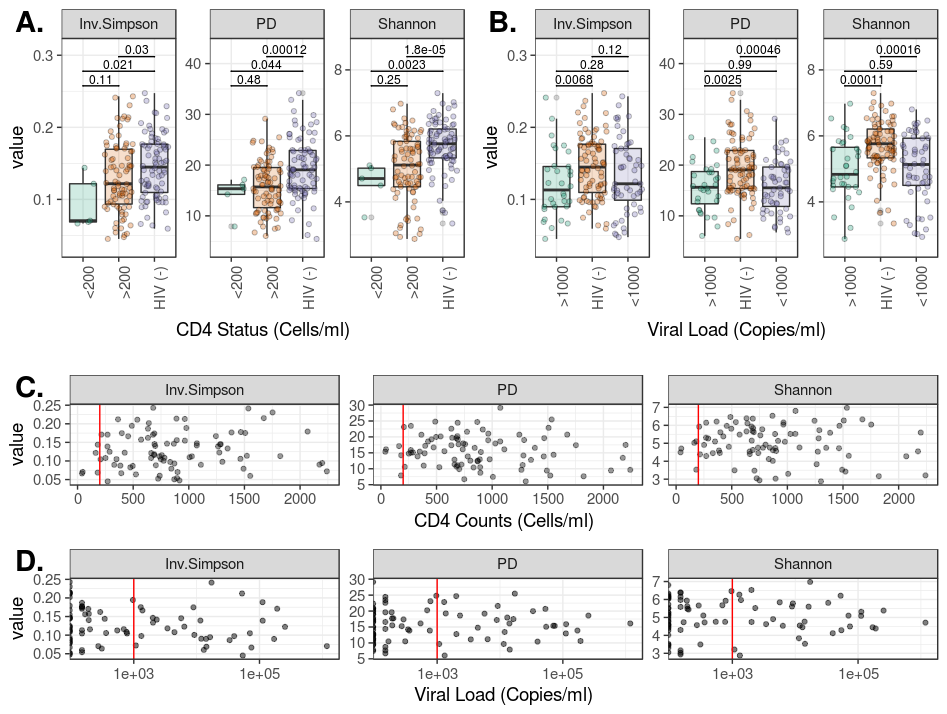

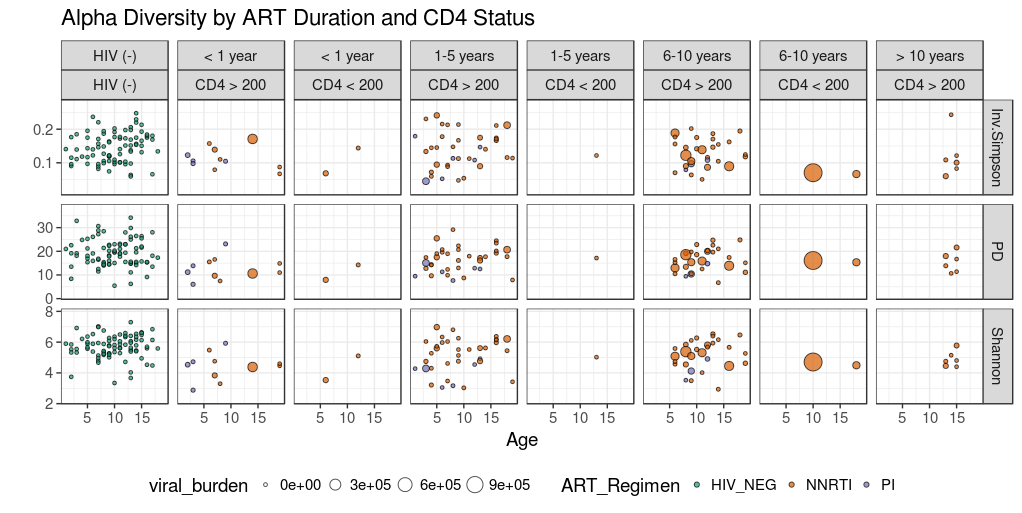


**Figure S2: Alpha diversity is not correlated with ART duration, age or immune status.** The top set of column facets groups by ART duration, and the bottom set of facets groups by CD4 status. The alpha diversity metrics are given in the faceted rows.
